# Expression of EFR3A in the Mouse Cochlea during Degeneration of Spiral Ganglion following Hair Cell Loss

**DOI:** 10.1371/journal.pone.0117345

**Published:** 2015-01-26

**Authors:** Chen Nie, Haixia Hu, Chenling Shen, Bin Ye, Hao Wu, Mingliang Xiang

**Affiliations:** 1 Department of Otolaryngology & Head and Neck Surgery, Xinhua Hospital, Shanghai Jiao Tong University School of Medicine, Shanghai, China; 2 Ear Institute, Shanghai Jiao Tong University School of Medicine, Shanghai, China; Harvard University, UNITED STATES

## Abstract

Retrograde degeneration of spiral ganglion cells in the cochlea following hair cell loss is similar to dying back in pathology. The *EFR3A* gene has recently been discovered to be involved in the pathogenesis of dying back. The relationship of EFR3A and spiral ganglion degeneration, however, was rarely investigated. In this study, we destroyed the hair cells of the mouse cochlea by co-administration of kanamycin and furosemide and then investigated the EFR3A expression during the induced spiral ganglion cell degeneration. Our results revealed that co-administration of kanamycin and furosemide quickly induced hair cell loss in the C57BL/6J mice and then resulted in progressive degeneration of the spiral ganglion beginning at day 5 following drug administration. The number of the spiral ganglion cells began to decrease at day 15. The expression of EFR3A increased remarkably in the spiral ganglion at day 5 and then decreased to near normal level within the next 10 days. Our study suggested that the change of EFR3A expression in the spiral ganglion was coincident with the time of the spiral ganglion degeneration, which implied that high expression of EFR3A may be important to prompt initiation of spiral ganglion degeneration following hair cell loss.

## Introduction

Recent studies have shown that cochlear hair cell regeneration in mammals is not impossible [1, 2]. Besides hair cell regeneration, regenerated hair cells must re-establish effective contact with the central auditory system for hearing restoration. As an important part of the human auditory system, spiral ganglion cells (SGCs) are responsible for accurately transmitting auditory stimulus from the hair cells to the cochlear nucleus. In addition, the efficiency of cochlear implants is also thought to be associated with the number of surviving SGCs. Preservation of SGCs, therefore, is essential for hearing rehabilitation [3].

Retrograde degeneration of SGCs in the cochlea following hair cell loss is similar to “dying back” in pathology. Dying back is a chronic process of nerve degeneration which occurs gradually from distal to proximal in neurodegenerative disorders such as Alzheimer’s disease [4]. Many previous studies [3, 5, 6] showed that following hair cell loss the cochlear nerve endings disappeared from the basilar membrane, and that weeks or months later the degeneration of the spiral neuron cell body occurred and progressively developed. Some researchers proposed that dying back might actually be active regulation determined by the axon itself internally [7, 8], and that axonal degeneration could result in neuronal death [9]. The *EFR3A* gene has recently been discovered to be involved in the pathogenesis of dying back. It encodes a unique membrane-bound protein with significant homology to DAG lipase. The EFR3A protein is a serine-aspartate-histidine catalytic triad consisting of 819 amino acids with a molecular weight of 92.6kD [10]. Its Drosophila homolog RBO played an important role in the maintenance of Drosophila sensory nerve function and in the degeneration of organic structure [11–13]. RBO is localized predominantly in presynaptic boutons and plays an essential role in neurotransmitter release and in mediating synaptic vesicle fusion [12]. It is required for bulk endocytosis and macropinocytosis in both neuronal and non-neuronal cells [13]. Moreover, EFR3A might be involved in auditory remodeling and immediate reaction to deprivation of acoustic signaling [10]. Based on these data, we hypothesized that EFR3A is involved in cochlear SGC degeneration following hair cell destruction. To test this hypothesis, we destroyed the mouse cochlear hair cells by co-administration of kanamycin and furosemide and then investigated the EFR3A expression during the induced SGC degeneration.

## Materials and Methods

### Subjects

Sixty-four healthy C57BL/6J adult mice (6 to 8 weeks old) were raised in an animal laboratory with free access to food and water. All animals were kept under standard laboratory conditions. The animals used in this study were cared for in strict compliance with the principles of the Institute of Laboratory Animals, Shanghai TongJi University School of Medicine. The protocol was approved by the Animal Use and Care Committee of Shanghai TongJi University School of Medicine (License No. 2009–0022, Shanghai). All surgery was performed under ketamine and xylazine anesthesia, and all efforts were made to minimize suffering.

### Kanamycin and furosemid treatment

All animals were randomly assigned to either a saline-treated or experimental group (8 animals in each group). Kanamycin (Sigma, USA) at a concentration of 75 mg/ml and furosemide (Tianjin Pharmaceutical Group, Tianjin, China) at a concentration of 10 mg/ml were freshly prepared before use. All experimental animals were administered first with a subcutaneous injection of kanamycin (1 mg/g) and then with an intraperitoneal injection of furosemide (0.4 mg/g) 30–45 minutes after [14]. The experimental animals were grouped as 1 day, 5 days, 15 days, 30 days and 60 days after injection. The control animals were grouped as 1 day, 30 days and 60 days after injection with the same dose of saline.

### Auditory brainstem response testing

Mice were prepared for auditory brainstem response (ABR) testing after anesthesia with a celiac injection of ketamine (100 mg/kg) and xylazine (10 mg/kg). ABR testing was recorded using a MEB-3102 physiological response recorder (Nihon Kohden, Tokyo, Japan). Pin electrodes were inserted into the mastoid prominence subcutis as the reference electrodes, into the thigh as the ground electrodes, and into the vertex as the active electrodes. Test stimuli were 4, 8, 12, and 16 kHz tone bursts generated using Tucker Davis Technologies hardware and software (SigGen) and presented at a rate of 21 times per second, filtered (100–3,000Hz).

### Preparation of cochlear tissue

Immediately following the ABR test, the mice were euthanized with an overdose of ketamine (200 mg/kg, intraperitoneal injection) and xylazine (20 mg/kg, intraperitoneal injection). The bullae were dissected out of the mice and the cochleae were removed. The apex of the cochlea, the round window and the oval window were punctured, followed by perfusion of 4% paraformaldehyde in 0.1 mmol/L phosphate-buffered saline (PBS pH 7.5) through the round window at 4°C overnight. The cochleae were then decalcified in 10% EDTA at room temperature for 7–8 days.

### Hair cell counting

The basilar membrane was dissected and cut into two (apical and basal) segments under a dissecting microscope. The tissue was blocked with 3% bovine serum albumin (BSA) in PBS containing 0.01% Triton-X-100 for 1 hour at room temperature, then incubated in a solution containing primary antibody for Myosin VIIa (Proteus BioSciences, CA, USA) diluted 1:300 at 4°C overnight. After being washed three times for a total of 10 minutes in PBS, the tissue was incubated with secondary antibody (1:400) (Alexa fluor molecular probe 594; Invitrogen) for 1 hour at room temperature. The tissue was washed with PBS and mounted on slides with antifade solution. Images were captured under a fluorescent microscope (Leica, Berlin, Germany).

### SGC density counting and transmission electron microscopy

Samples for the transmission electron microscopy were prepared as follows. Cochlear tissues were fixed with 2.5% glutaraldehyde for at least 2 hours, washed in 0.1 M PBS three times, immersed in 1.0% OsO4 for 2–3 hours at room temperature for further fixation, then washed again. After dehydration in a graded series of ethanol solutions and acetone, the samples were embedded in Eponate 12. Sections approximately parallel to the modiolus were taken at five to six successive levels to enable all turns of the cochlear spiral to be examined. At each level, an initial 1 mm-thick semi-thin section was prepared and stained in toluidine blue for light microscopy. Thin sections (50–60 nm) were cut using an ultramicrotome LKB-I and stained sequentially with 3% uranyl acetate and lead citrate, and were examined with a transmission electron microscope (Philips CM-120; Philips).

For SGC counting, every fifth midmodiolar was mounted on slides and stained with toluidine blue for estimating SGC numbers from apical and basal turns, with 5 cochleae per group. To measure the cross sectional area of Rosenthal’s canal, we used Image J software. SGC density was calculated by dividing the number of perikarya by the cross-sectional area. The group averages for SGC density was based on five animals per group, computed separately for the apical and basal turns.

### Fluorescent immunohistochemistry

Following several rinses in 0.1M PBS, decalcified cochleae were preceded with dehydration in 10%, 20%, and 30% sucrose in sequence until the tissue sank. Next, the cochleae were embedded in OCT compound entirely and then frozen at -20°C. Frozen sections (6μm) were obtained parallel to the modiolus with a freezing microtome. Cryosections were washed in PBS for 30 minutes, and then incubated with a blocking solution containing 3% BSA in PBS for 1 hour at room temperature. Sections were incubated in the mixed dilute primary antibody (1:500 mouse anti-TUJ1 antibody and 1:200 dilution of rabbit anti-EFR3A (Sigma) antibody) at 4°C overnight in a humidified chamber. After the sections were washed in PBS three times, they were incubated in 1:400 FITC-conjugated donkey anti mouse and 1:400 Texas red-conjugated goat anti rabbit secondary antibody diluted in BSA Blocking reagent for 1 hour at room temperature in a dark humidified chamber. The sections were washed again, labeled with DAPI (4′,6-diamidino-2-phenylindole), and mounted using Prolong-Gold Anti-fade reagent. The control sections were incubated in the parallel process without primary antibodies. Images were captured using a fluorescent microscope (Leica, Berlin, Germany).

### Western blotting

Four cochlear organs were isolated, digested and centrifuged from each group. The supernatant was mixed with loading buffer, boiled for 10 minutes, and stored at -20°C for later analysis. Samples were run on 8% SDS-PAGE gels for 60 minutes at 120 V and transferred to immobilon polyvinydifluoride membranes (Millipore, Billerica, MA, USA) using 0.3 A for 70 minutes. Membranes were blocked in 5% nonfat dry milk in TBS-T for 1 hour at room temperature, incubated with antibodies against TUJ1 (Covance, 1:1000) and EFR3A (Sigma, USA; 1:300) for 1 hour at room temperature and then overnight at 4°C, washed three times for 10 minutes in TBS-T, and incubated in HRP-conjugated secondary antibody (goat anti-rabbit IgG or rabbit anti-mouse IgG) for 1 hour at room temperature. After washed again, the immunoreactive bands were visualized by enhanced chemiluminescence according to manufacturer’s instructions with Kodak X-OMAT LS film (Eastman Kodak, Rochester, NY, USA).

### Statistical analysis

All statistical analyses were performed using SPSS version 13.0 software. ABR, hair cell counting, and SGC density data were presented as the mean ± standard error and analyzed using one-way ANOVA. A p-value of less than 0.05 was considered to be statistically significant.

## Results

### ABR thresholds

The ABR threshold of three control groups mice at pure tones of 4 kHz, 8 kHz, 12 kHz, and 16kHz frequencies were 45.9 ± 1.5 dB, 40.0 ± 1.8 dB, 43.3 ± 3.1 dB, and 45.0 ± 3.7 dB respectively, with no significant changes (p >0.05). After administration of kanamycin and furosemide, the ABR thresholds of the experimental group increased significantly on the first day to 67± 2.8 dB, 57±2.5 dB, 69±3.0 dB and 76±2.5 dB at 4 kHz, 8 kHz, 12 kHz and 16 kHz, respectively. There were no significant increases in ABR thresholds at 5, 15, and 30 days following injection at any frequency tested. By 60 days after injection, the ABR thresholds increased slightly to 85±1.5 dB, 87±1.7 dB, 86±1.6 dB and 88±1.1 dB at 4 kHz, 8 kHz, 12 kHz and 16 kHz, respectively. The results showed that the drug administration caused irreversible deafness during the observation period and implied that cochlear hair cells were likely to suffer complete damage.

### Hair cell loss

**Fig 1:**
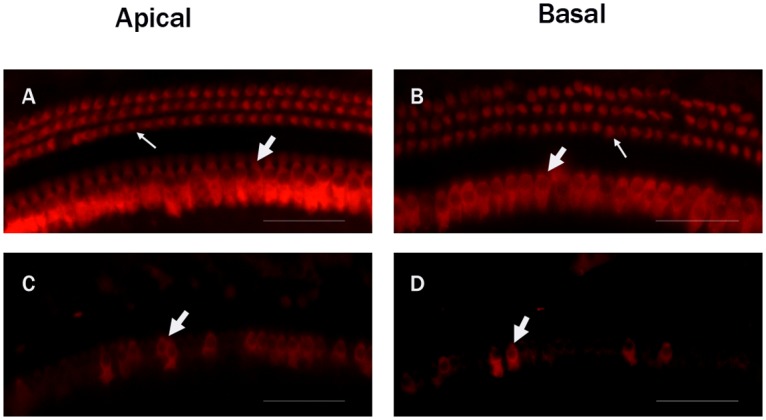
Fluorescent immunohistochemistry images of myosin VII7a-labeled hair cells in the cochleae of the C57BL/6J mice. A: The apical turn in the control group. B: The basal turn in the control group. Three rows of outer hair cells (Thin white arrow) and one row of inner hair cells (Thick white arrow) in the control group were complete and arranged orderly. C: The apical turn in the experimental group at the first day following drug administration. D: The basal turn in the experimental group at the first day. Most of the outer hair cells and part of the inner hair cells in the experiment groups were destroyed. Scale bar = -25μm.

**Fig 2:**
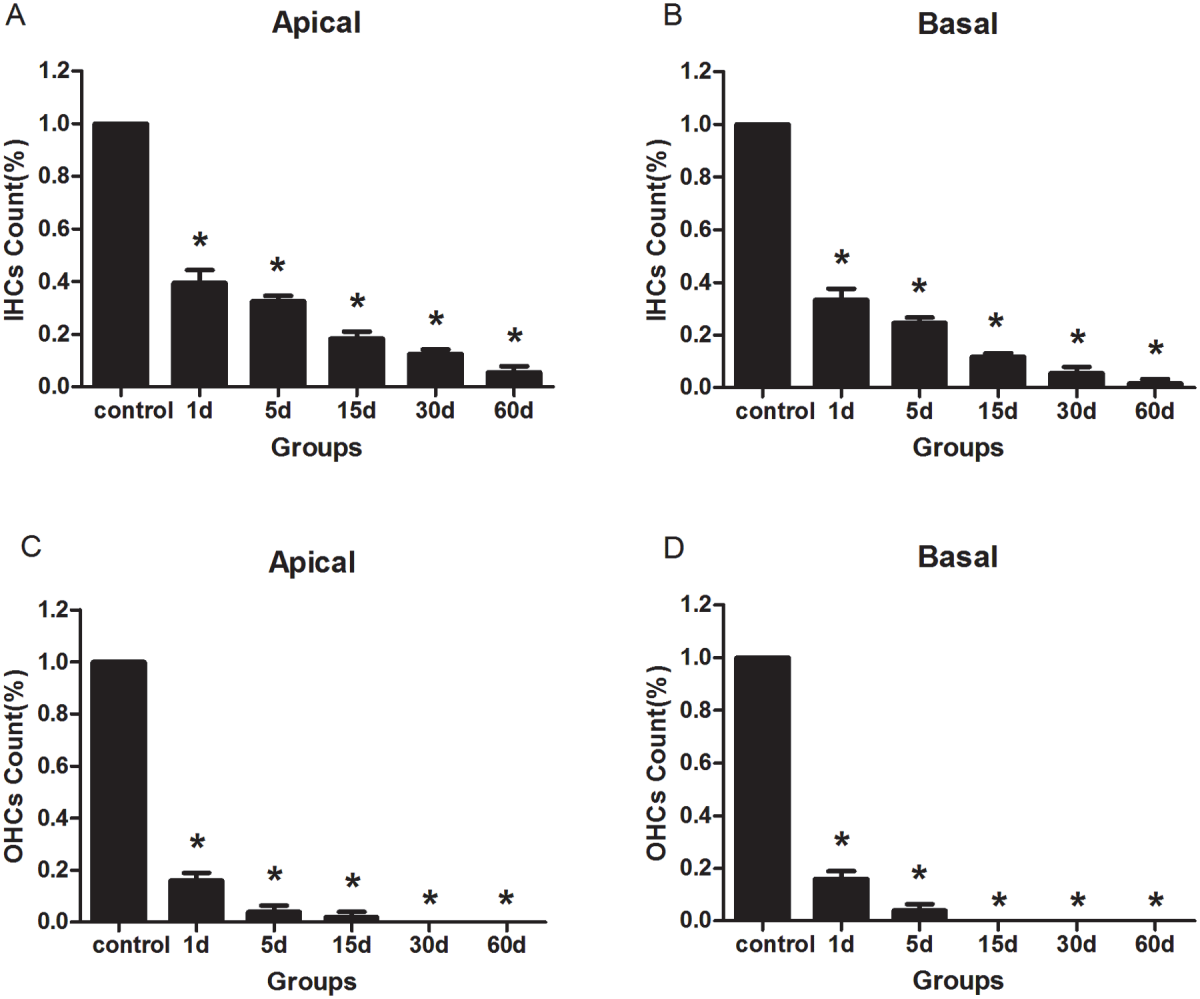
The changes of hair cells in the cochleae of the animals treated with kanamycin and furosemide. A: Inner hair cells counts from the apical turn. B: Inner hair cells counts from the basal turns. C: Outer hair cells counts from the apical turns. D: Outer hair cells counts from the basal turns. Four animals were included in each group. In each cochlea two representative locations were counted. It showed that the drug administration successfully destroyed almost all hair cells in the cochlea. Asterisks indicated significant (*P* <0.05) difference between the experimental and the control groups.

Immunofluorescence staining by anti-Myosin Ⅶa antibody was used to observe the cochlear hair cells. The control groups showed no difference in the appearance of the hair cells. Three rows of outer hair cells and one row of inner hair cells were arranged orderly (Figs. 1A, B). At the first day after the injection of kanamycin and furosemide, the majority of the outer hair cells and part of the inner hair cells in the cochlea were destroyed (Figs. 1C, D, 2). The impairment of hair cells continued. All outer hair cells disappeared at the 30th day, and by the 60th day most of the inner hair cells were damaged (Fig. 2). The results were consistent with previous morphological findings by Hirose K et al [15]. In addition, compared with apical hair cells, the basal turn region exhibited a more obvious loss at any time points observed (Fig. 2). The morphological results showed that the drug administration successfully destroyed almost all hair cells in the cochlea.

### SGC degeneration

**Fig 3:**
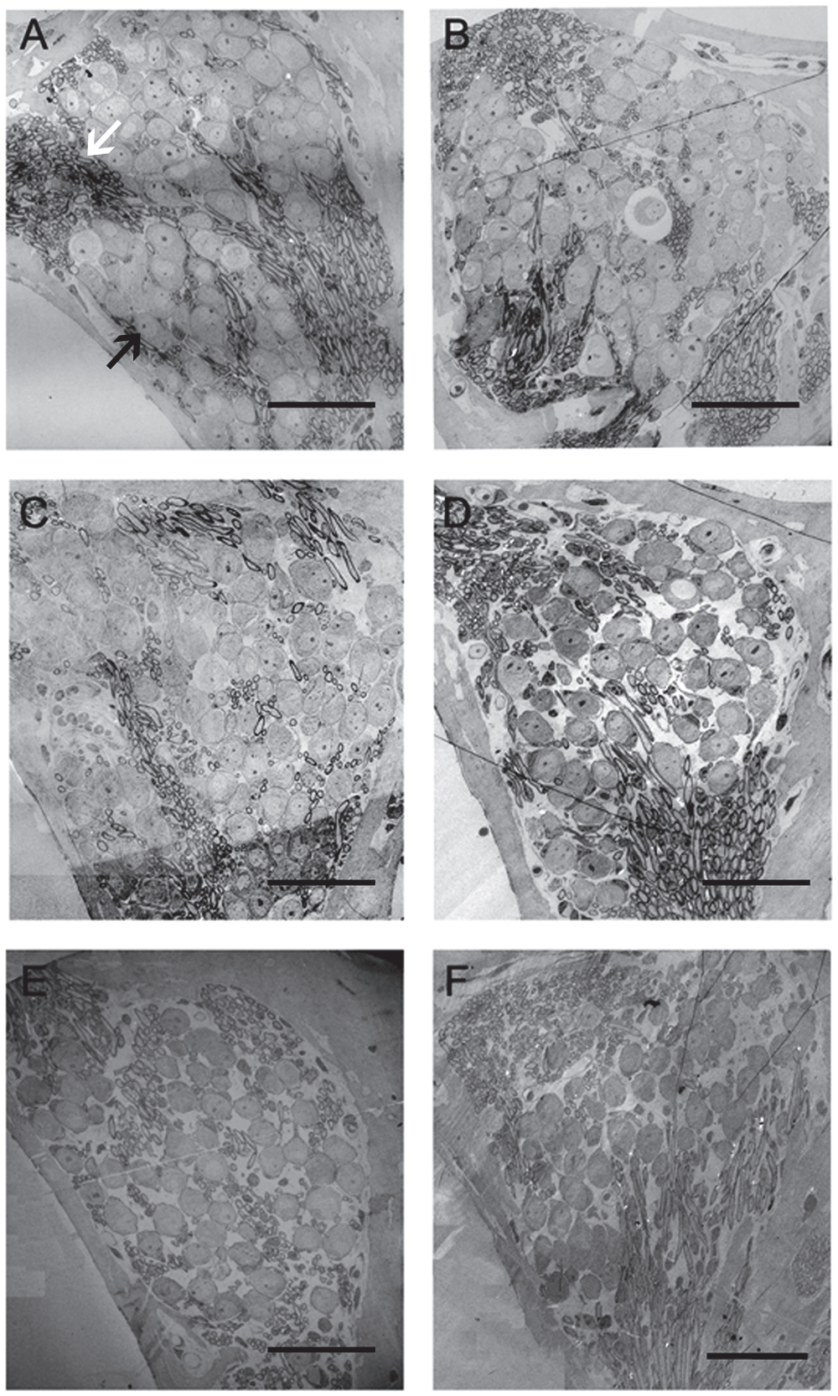
Low power transmission electron micrograph of the SGCs taken from the cochleae of the animals treated with kanamycin and furosemide. A: The SGCs in the cochlea from the control group. The SGCs were arranged tightly. B: The SGCs in the experimental group 1 day following the end of drug administration. Compared with the control group, no reduction in SGC number occurred. C: The 5th-day experimental group. Also no significant reduction in SGC number occurred. D: The 15th-day experimental group. Compared with the control group, a significant reduction in SGC number appeared. E: The 30th-day experimental group. SGC loss was progressive. F: The 60th-day experimental group. SGC loss was further aggravated. Black arrow: spiral ganglion cells; white arrow: myelinated nerve fibers. Scale bar = 50μm.

**Fig 4:**
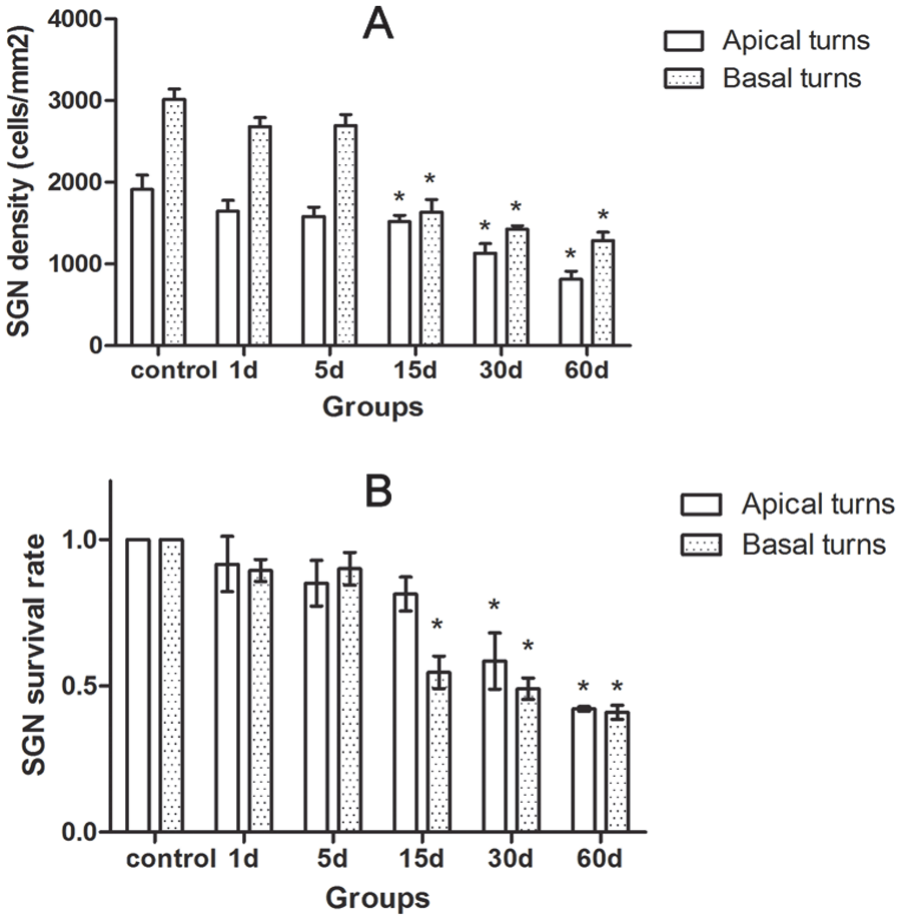
A: The changes of SGC density in the cochleae of the animals treated with kanamycin and furosemide. B: Changes of SGC survival rate in the cochleae of the animals treated with kanamycin and furosemide at survival time. It showed that the SGC degeneration in the cochlea following hair cell loss was progressive. Asterisks indicated significant (*P* < 0.05) difference existed compared to the control group.

Similar to the previous report [16], our results showed that the number and the density of the SGCs of the experiment animals did not notably decrease until the 15th day following injection (Figs. 3D, 4). The SGCs degenerated rather progressively (Figs. 3E, F, 4). The SGCs in the basal turns of the cochlea exhibited a more rapid onset of neuronal loss than in the apical turns, and by the 60th day following injection of kanamycin and furosemide the relative losses were similar (Fig. 4).

**Fig 5:**
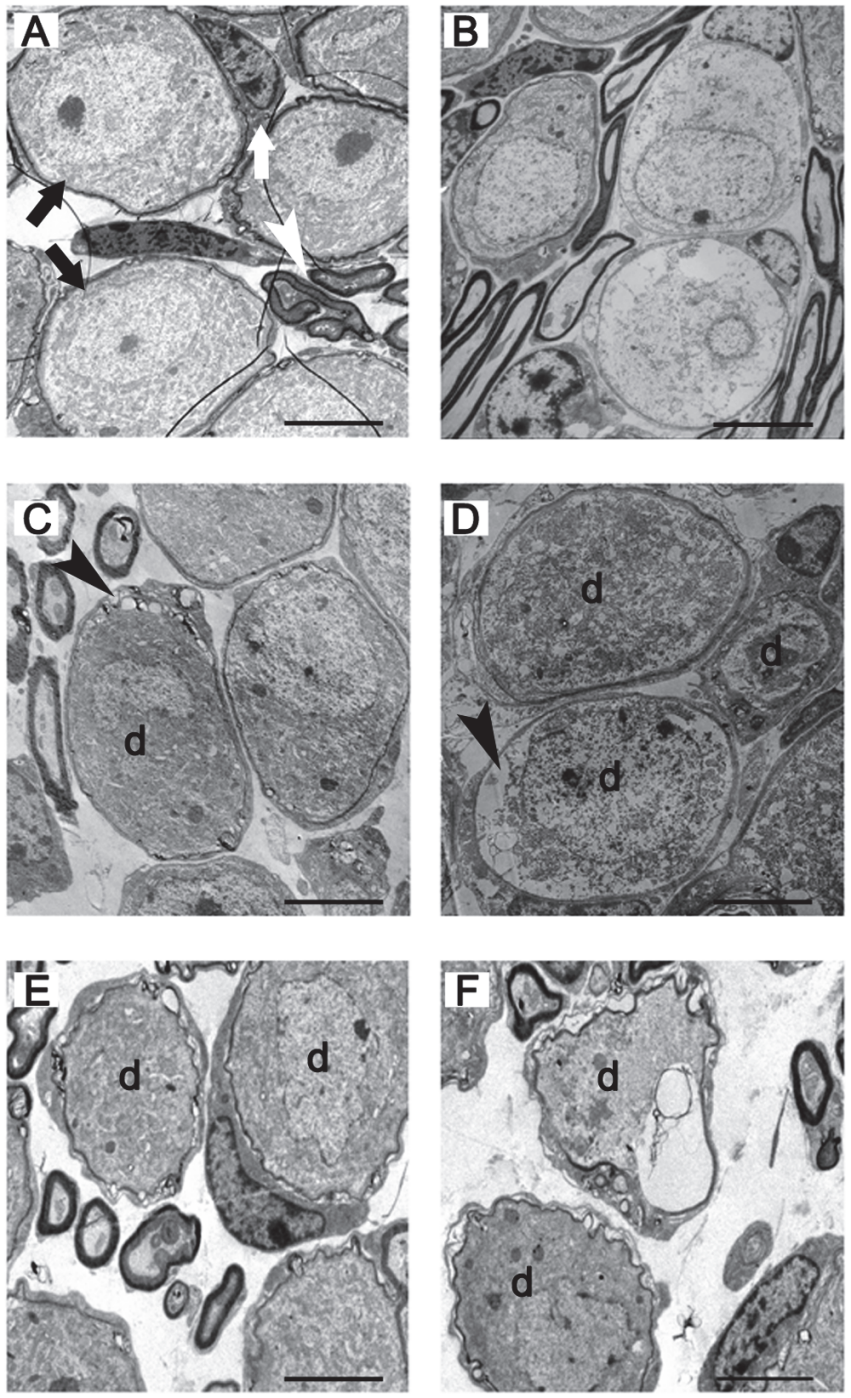
Transmission electron micrograph of the SGCs taken from the basal turns of the cochleae in the animals treated with kanamycin and furosemide. A: The SGCs in the cochlea from the control group. B: The SGCs in the experiment group 1 day following the end of drug administration. Compared with the control group, no abnormal changes occurred. C: The 5th-day experiment group. The abnormal changes of SGCs appeared. In the SGCs, cytoplasmic vacuoles gathered at the edge of the cytoplasm, with a few swelling and scattered mitochondria and vague nucleoli. D: The 15th-day experiment group. SGC degeneration was progressive. E: The 30th-day experiment group. SGC degeneration was further aggravated. Balloon-like and vacuole-like spaces appeared around the perikarya of SGCs. Abnormal, partially collapsed or folded myelin sheaths were seen. F: The 60th-day experiment group. SGC degeneration was more severe. Black arrow: spiral ganglion cells; white arrow: Schwann cell; white arrowhead: myelinated nerve fibers; black arrowhead: cytoplasmic vacuoles; d: degenerated spiral ganglion neurons. Scale bar = 5μm.

The ultrastructural morphology of the C57BL/6J mouse SGC degeneration following hair cell loss was rarely reported. In our study, no difference was evident in the ultrastructural morphology of SGCs between the control (Fig. 5A) and the experiment animals 1 day following injection (Fig. 5B). By the 5th day, however, the cytoplasmic vacuoles of the spiral ganglion neuronal cells of the experiment animals gathered at the edge of the cytoplasm with a few swelling, scattered mitochondria and vague nucleoli (Fig. 5C). Afterwards more and more spiral neuronal cell bodies became swollen and larger, and the degenerated organelles increased (Figs. 5D, E, F). Following the progress of SGC degeneration, the decrease of the number of the SGCs became evident (Figs. 5E, F). Many Schwann cells lost their normal shape (Figs. 5E, F).

### The expression of EFR3A in the spiral ganglia

**Fig 6:**
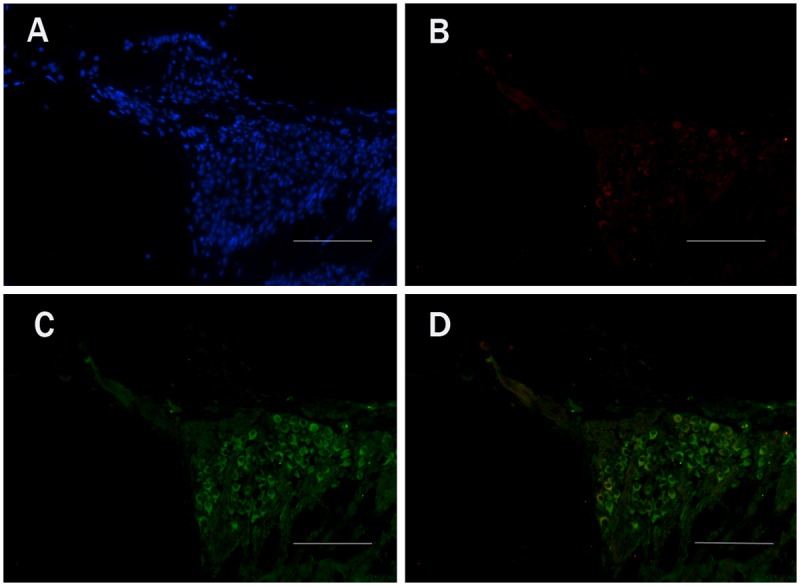
Fluorescent immunohistochemistry images of EFR3A expression in the basal turns of cochlea from the control group. A: DAPI staining. B: anti-EFR3A staining. C: TUJ1 staining. D: Co-labeling images of TUJ1 and EFR3A. It showed that EFR3A was localized predominantly in the SGCs of the cochlea. Scale bar = 50μm.

**Fig 7:**
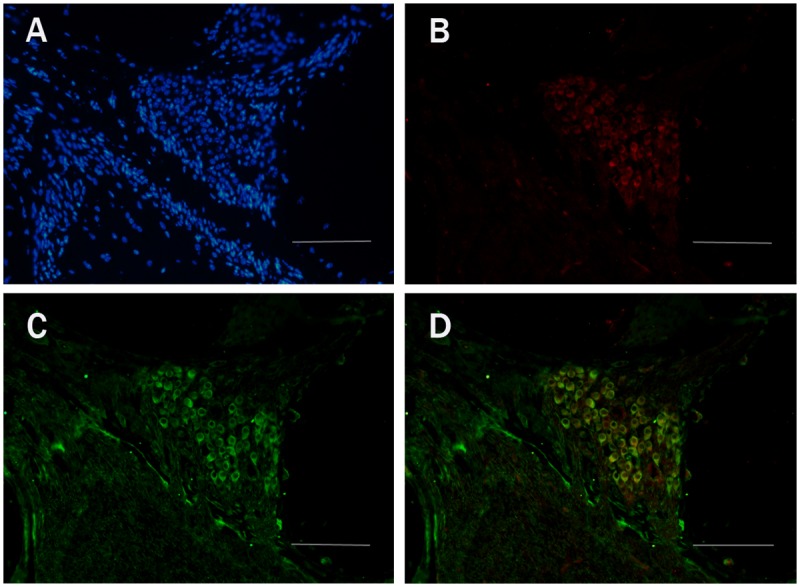
Fluorescent immunohistochemistry images of EFR3A expression of the SGCs in the basal turns of cochlea from the experiment animals at the 5th day following drug administration. A: DAPI staining. B: anti-EFR3A staining. C: TUJ1staining. D: Co-labeling images of TUJ1 and EFR3A. Scale bar = 50μm.

We used fluorescent immunohistochemical staining to identify which types of cells expressed EFR3A in frozen sections of the mouse cochleae. The spiral ganglion neurons were co-labeled with the antibody to TUJ1, a SGC specific marker. Our results demonstrated that the EFR3A was localized predominantly in the basal turns, but not in the apex turns (data not shown), of the spiral ganglion neurons of the cochlea (Fig. 6D, 7D). In the control group, EFR3A displayed low and sparse expression in the SGCs (Fig. 6). In the experiment group, the expression of EFR3A was evidently increased at the 5th day following injection (Fig. 7).

**Fig 8:**
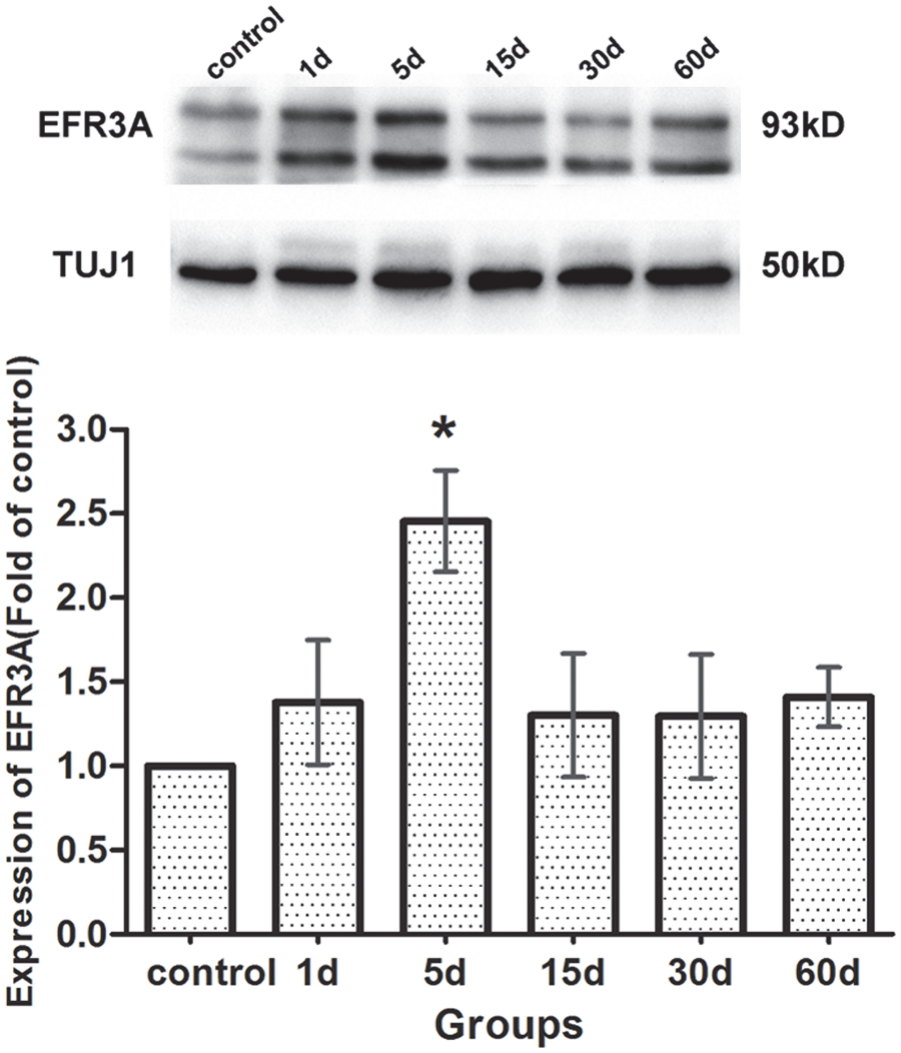
The changes of EFR3A expression in the organ of Corti in the animals treated with kanamycin and furosemide by western blotting. TUJ1 protein, with a molecular weight of 50 KD, was identified as an internal reference. The molecular weight of EFR3A was 93 KD. At the 5th day following drug administration, EFR3A expression was remarkably increased in the cochleae of the experiment animals. By the 15th day the EFR3A expression decreased to nearly normal level. All the six samples were run on the same gel adjacent to one another. The TuJ1 and EFR3A bands were obtained from the same stripped blot. The histogram was based on all four experiments of western blotting, and the Image Lab software and ANOVA statistical method were used. Asterisks indicated significant (*P* <0.05) difference compared with the control group.

We further compared the EFR3A expression in the cochleae between the control and the experimental animals 1, 5, 15, 30, and 60 days following drug administration. Western blotting analysis was performed using the TUJ1 protein as the internal control. Our results (Fig. 8) showed that there was no significant difference in the expression of EFR3A in the cochleae between the control and the experiment animals on the first day following injection. At the fifth day, a remarkable increase of EFR3A expression occurred in the cochleae of the experiment animals. Afterwards, the EFR3A expression decreased to nearly normal level by the 15th day. Some non-significant fluctuations of the EFR3A expression were observed in the cochleae in the experimental groups of 30 days and 60 days.

## Discussion

In the present study, we adopted the protocol of kanamycin-furosemide co-administration to destroy cochlear hair cells in C57BL/6J mice and to explore the degeneration of spiral ganglion neurons in the cochlea. At the first day after drug administration, mice showed obvious elevations of ABR thresholds compared to the controls and this elevation further increased with time. Accompanying with the functional impairment, the experimental animals displayed obvious morphological damage in the majority of the outer hair cells and part of the inner hair cells at the first day after administration. The damage of the hair cells continued. By the 30th day all the outer hair cells disappeared and by the 60th day most of the inner hair cells were destroyed. These results demonstrated that the combined injection of kanamycin and furosemide successfully destroyed almost all hair cells in the cochleae of the mice, and laid the foundation for the occurrence of cochlear spiral ganglion neuron degeneration.

The morphological changes of the SGCs responding to the drug-induced deafness varied depending on the species and dosages used [5, 6]. Kong et al.[5] observed that the effect of the kanamycin on hair cells, SGCs and Schwann cells was progressive in the cochleae of the guinea pigs injected with kanamycin for 7 days, and that the impairment of outer hair cells maybe an important factor leading to SGC degeneration. McFadden et al.[6] reported that the SGC loss occurred in the cochleae of chinchillas 30 days after hair cells damage induced by the co-administration of gentamicin and ethacrynic acid, and that the degeneration of the SGCs developed gradually. Nourski et al.[16] reported that the decrease of the SGC number in the cochleae of the guinea pigs started at 14 days after the co-injection of kanamycin and furosemide, which was similar to our finding that the decline of the SGCs density in the C57BL/6J mice began at the 15th day after hair cell loss. However, although the SGC loss did not occur until the15th day following drug administration, our data showed that the abnormal morphological changes of the SGCs appeared at the 5th day already including slight shape changes, scattered small vacuoles margination and a few swelling mitochondria in some SGCs. With time, the degeneration of the SGCs proceeded progressively in the cochleae in the experiment animals. These morphological results indicated that the SGC degeneration induced by the hair cell loss in the cochlea was to certain extent similar to dying back.

It is generally thought that hair cells are required for SGC survival and loss of inner hair cells results in loss of the SGCs [3, 17–19]. In our study, we also observed that the reduction of number of SGCs appeared later than the impairment of hair cells in the cochlea. Meanwhile, we also found that the decrease of the number of spiral ganglion neurons appeared to be unsynchronized with the degree of impairment of the hair cells. Although the decline of the number of SGCs in the cochlea was apparent on the 15th day after co-injection, when almost all outer hair cells and 80% inner hair cells were injured, the number of spiral ganglion neurons was further (though slightly) reduced at the 60th day when nearly all hair cells disappeared. Some researchers believed that the remaining spiral neural cells may not only relate with the surviving hair cells but also with the surviving Schwann cells [20, 21]. However, the results in our study showed that the Schwann cells in the cochleae also suffered severe damage with time after drug administration. Based on our observation, we believed that there might be other important factors to affect the cochlear spiral ganglion neuron degeneration in addition to impairment of hair cells and Schwann cells.

SGC degeneration in the cochlea following hair cell loss is similar to dying back in pathology. Some researchers reported that EFR3A might be involved in the process of dying back [11–13]. In this study, we observed EFR3A expression in the mouse cochlear spiral ganglion neurons by immunofluorescence staining. We found that the EFR3A expression was remarkably increased at the 5th day after co-administration. The time of the high EFR3A expression was coincident with the time of the appearance of the abnormal ultrastructural changes of the SGCs. These results indicated that EFR3A protein might play a significant role in SGC degeneration in the cochlea following hair cell loss. Munemoto Y et al.[10] reported that EFR3A expression in the lateral superior olive was significantly increased immediately following malleus removal and cochlear ablation. That study, however, did not investigate the changes of EFR3A protein expression in the cochleae and in the auditory system in the prolonged period. In the present study, we found that the EFR3A expression in SGCs remained high only shortly. By the 15th day after administration the expression was decreased to normal. No obvious changes were found in the experiment animals afterwards. Given the fact that the high expression of EFR3A in the cochlear spiral ganglion neurons mainly occurred in the early degeneration of the SGCs, we believe that EFR3A may play an intermediary role in inducing the degeneration of the SGCs in the cochlea.

In the present study, the expression of EFR3A can be hardly detected by immunofluorescence staining in the apical turn of cochlea either in the control groups or in the experiment groups. For the control group, the interpretation may be given as follows: 1) in SGCs of the normal animals the EFR3A expression was low and sparse; 2) compared with the basal turn, the number of SGCs in the apical turn was much less, therefore the detection was difficult. For the experiment group, there are three possible explanations: 1) the degeneration of SGCs induced by hair cell loss starts from the basal toward the apical turn. At the 5th days after administration, the degeneration of the SGCs initiated in the basal turns but not in the apical turns. Therefore the expression of EFR3A increased significantly in the basal turn but not in the apical turn; 2) in the experiment groups of 15, 30 and 60 days, the SGC loss was evident in the apical turns already, so it was difficult to detect the EFR3A expression; 3) the sustaining time of the high expression of EFR3A was short. We may miss the time that EFR3A expression was high in the apical turn.
